# Mechanisms of peptide sex pheromone regulation of conjugation in *Enterococcus faecalis*

**DOI:** 10.1002/mbo3.492

**Published:** 2017-05-19

**Authors:** Yuqing Chen, Arpan Bandyopadhyay, Briana K. Kozlowicz, Heather A. H. Haemig, Albert Tai, Wei‐Shou Hu, Gary M. Dunny

**Affiliations:** ^1^ Department of Microbiology and Immunology University of Minnesota Minneapolis MN USA; ^2^ Department of Chemical Engineering and Materials Science University of Minnesota Minneapolis MN USA; ^3^ Tufts University Boston MA USA; ^4^ Present address: Cargill Biotechnology R&D Plymouth MN USA; ^5^ Present address: Department of Chemistry Gustavus Adolphus College St. Peter MN USA

**Keywords:** antibiotic resistance, bacterial transcription, cell signaling, co‐repressor, gene transfer, gram positive bacteria, protein‐nucleic interaction, RRNPP transcription factor

## Abstract

In many gram positive bacteria, horizontal transfer and virulence are regulated by peptide‐mediated cell‐cell signaling. The heptapeptide cCF10 (**C**) activates conjugative transfer of the *Enterococcus faecalis* plasmid pCF10, whereas the iCF10 (**I**) peptide inhibits transfer. Both peptides bind to the same domain of the master transcription regulator PrgX, a repressor of transcription of the *prgQ* operon encoding conjugation genes. We show that repression of *prgQ* by PrgX tetramers requires formation of a pCF10 DNA loop where each of two PrgX DNA‐binding sites is occupied by a dimer. **I** binding to PrgX enhances *prgQ* repression, while **C** binding has the opposite effect. Previous models suggested that differential effects of these two peptides on the PrgX oligomerization state accounted for their distinct functions. Our new results demonstrate that both peptides have similar, high‐binding affinity for PrgX, and that both peptides actually promote formation of PrgX tetramers with higher DNA‐binding affinity than Apo‐PrgX. We propose that differences in repression ability of PrgX/peptide complexes result from subtle differences in the structures of DNA‐bound PrgX/peptide complexes. Changes in the induction state of a donor cell likely results from replacement of one type of DNA‐bound peptide/PrgX tetramer with the other.

## 1 INTRODUCTION

Many bacteria coordinate multicellular behaviors using secreted signaling molecules for intercellular communication. While gram negative bacteria frequently employ N‐acyl homoserine lactones as extracellular signals, gram positive bacteria generally use oligopeptides (Waters & Bassler, 2005). Some oligopeptide signals interact with membrane‐bound histidine kinases and transmit information via two‐component signal transduction, while others are transported into the responder cell, where they bind to their cognate intracellular receptors to initiate a response (Chandler & Dunny, 2004; Dunny & Leonard, 1997).

The recently described RRNPP family of sensing proteins act as receptors for peptide signals controlling development, virulence, and horizontal gene transfer in gram positive bacterial pathogens (Declerck et al., 2007; Parashar, Aggarwal, Federle, & Neiditch, 2015; Rocha et al., 2012). This family of proteins includes Rap proteins (*Bacillus* aspartyl phosphate phosphatases), the *Bacillus* neutral protease regulator NprR and its orthologs, the pleiotropic regulator PlcR from *B. cereus* group, *Streptococcus* Rgg proteins, and the sex pheromone receptor proteins PrgX and TraA from *E. faecalis* (Cook & Federle, 2014; Declerck et al., 2007). The signaling peptides are synthesized as immature propeptides, secreted from the cell, and undergo proteolytic maturation during secretion. The mature peptides are internalized from the growth medium, and bind directly to their cognate RRNPP receptor. Except for the Rap phosphatase proteins, RRNPP proteins are transcription factors, whose activity is modulated by binding of their specific peptide signals (Cook & Federle, 2014; Declerck et al., 2007). While the RRNPP systems regulate numerous critical functions related to virulence and horizontal gene transfer in important pathogens, our understanding of the molecular mechanisms by which these peptide‐dependent gene regulatory circuits function is limited. Although amino acid sequence homology among RRNPP proteins is low, these proteins all have similar structures. The first‐identified members of this family of transcription factors are encoded by the enterococcal sex pheromone plasmids pAD1 and pCF10 (Clewell & Dunny, 2002; Clewell et al., 2014), and serve as models for the more recently discovered systems.

In *Enterococcus faecalis*, pCF10‐containing donor cells respond to a 7‐amino‐acid sex pheromone cCF10 (**C**‐ clumping‐inducing peptide, amino acid sequence LVTLVFV), typically produced by recipient cells (Antiporta & Dunny, 2002). The pheromone is imported into donor cells and binds to the master transcription regulator PrgX (Kozlowicz et al., 2004, Kozlowicz et al., 2006; Leonard, Podbielski, Hedberg, & Dunny, 1996; Shi et al., 2005). Pheromone binding to PrgX relieves repression of the pCF10 conjugation genes. **C** is chromosomally encoded and pCF10 has evolved two mechanisms to avoid self‐induction of donors by endogenous pheromone (Dunny, 2013). The plasmid‐encoded membrane protein PrgY reduces the level of pheromone activity produced by donors (Chandler, Flynn, Bryan, & Dunny, 2005). Residual pheromone activity is neutralized by a 7‐amino‐acid inhibitor peptide iCF10 (**I**‐ amino acid sequence AITLIFI), which is encoded by the *prgQ* gene of pCF10 (Nakayama, Ruhfel, Dunny, Isogai, & Suzuki, 1994). Recently it has been shown that **I** has two distinct roles. In uninduced donor cultures, **I** accumulates with population density and serves as a classical quorum sensor of donor density; thus at high population density, donor cells are poorly induced by exogenous pheromone (Chatterjee et al., 2013). In addition, **I** plays an essential role in returning donors to the uninduced state following an induction by **C**. The *prgQ* gene encoding **I** is located at the 5′ end of the long, pheromone‐inducible operon encoding conjugation genes.

(Hirt et al., 2005); induction thus leads to accumulation of **I** in the growth medium, which eventually overcomes the **C** signal and shuts off the response.

The **I** and **C** peptides are cleaved from precursors, secreted outside the cell and imported by the plasmid‐encoded PrgZ peptide‐binding protein and chromosomally encoded oligopeptide permeases (Leonard et al., 1996). The imported peptides compete for binding to PrgX (Kozlowicz et al., 2006). Structural analysis of PrgX/**C** and PrgX/**I** complexes showed that both peptides bind to the same cleft in the dimerization domain (Kozlowicz et al., 2006; Shi et al., 2005). However, the two peptides interact with different residues in the PrgX carboxy terminus. While all PrgX crystals examined to date contained tetramers, **C** binding caused PrgX carboxy‐terminal helix 17 to refold into a β‐duplex that covers **C**, while **I** stabilized a C‐terminal 10 residue β‐strand which serves as an interacting face promoting tetramer formation between pairs of dimers (Kozlowicz et al., 2006; Shi et al., 2005). Based on these data, it was suggested that peptide‐induced changes in the C‐terminus of PrgX could alter the protein oligomerization state in solution: **I** was predicted to stabilize a tetramer structure, whereas **C** binding was predicted to destabilize PrgX tetramers, favoring a dimer state in solution. No structural information about PrgX/DNA complexes is available, and prior to this report there was no direct evidence for how peptide binding affected PrgX oligomerization in solution.

**Figure 1:**
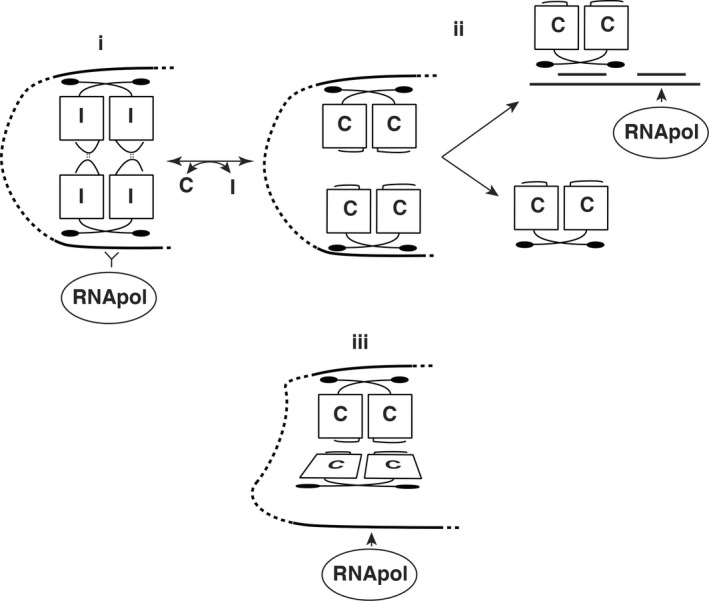
Models of PrgX function. Part i. shows a repressing complex of PrgX/I tetramers, where both XBSs are tightly bound by PrgX, preventing access RNA polymerase to the *prgQ* promoter. Part ii. depicts a previous working model for I is replacement by C in a tetramer, changing the C‐terminal structure of PrgX by moving a predicted tetramer‐stabilizing loop of the protein (Kozlowicz et al., 2006; Shi et al., 2005). Dissociation of the tetramer would weaken the DNA loop and favor PrgX dissociation from XBS2, allowing RNA polymerase to access the promoter. Part iii. Depicts the current working model, based on new results reported here. In this model, both peptides promote tetramer formation and looping, but the PrgX/C tetramer is distorted, placing torsional stress on the DNA loop structure reducing tight binding of PrgX to XBS2, and enabling RNA polymerase to compete more effectively for binding to the promoter. Conversion from i. to iii. occurs by replacement of one form of PrgX with another on the DNA rather than replacement of one peptide with the other in a preformed tetramer

The target of PrgX‐peptide‐mediated regulation is the *prgQ* promoter (*P*_Q_), which controls expression of the majority of factors involved in pCF10 conjugation. PrgX binds specifically to two operator sites (XBS1 and XBS2) in the *P*_Q_ region between *prgX* and *prgQ*. Because the lower‐affinity XBS2 site overlaps P_Q_, occupancy of XBS2 by PrgX could impede RNA polymerase binding to the promoter. Based on genetic and structural data, it was proposed that pairs of PrgX dimers could bind to XBS1 and XBS2, with the intervening DNA forming a loop that is stabilized by protein–protein interactions between the dimers bound to each operator site. Because **I** was predicted to stabilize PrgX tetramers, **I** should enhance repression of *P*_Q_ transcription (Figure 1i). On the other hand, if as previously predicted, **C** binding to PrgX destabilizes tetramers in solution (Figure 1ii), **C** should dissociate the DNA‐bound tetramer, disrupt the loop, and lead to dissociation of PrgX from XBS2, and increase transcription from *P*_Q_. We have used in vitro transcription assays to show that PrgX directly represses *P*_Q_ (Caserta et al., 2012). However, our previously published DNA‐binding assays and in vitro transcription experiments with purified PrgX did not examine the effects of two peptides on the biochemical activity of PrgX. Thus, it is still not known how these two similar peptides modulate PrgX activity differentially to result in opposite outcomes in cells carrying pCF10. In this paper, we confirm two major features of the model for PrgX regulation. Namely, that a DNA loop formed by pairs of interacting PrgX dimers bound to the two XBSs is required for repression of *prgQ* transcription, and that competition between PrgX and RNA polymerase for binding in the XBS2 region is critical for regulation. We also report the surprising results of experiments analyzing the affinity of **C** and **I** for PrgX, as well as the effects of each peptide on the DNA‐binding activities of PrgX, and on the PrgX oligomerization state in solution. We formulate a new model for the differential effects of operator‐bound PrgX/**C** versus PrgX/**I** complexes on *P*_Q_ expression. This model (Figure 1i, iii) incorporates new data indicating that both PrgX/**I** and PrgX/**C** complexes form tetramers that bind to the target operators with high affinity, but differences between the DNA/protein complexes render the **C**‐containing complex less able to compete with RNA polymerase for binding to the *P*_Q_ region. The data also suggest that transitions between the repressed and de‐repressed states in donor cells result from replacement of DNA‐bound PrgX tetramers of one form with the other rather than conversion of tetramers by displacement of one peptide with the other. These new results have important implications for the pathways by which donor cells respond to these two signaling molecules.

## 2 EXPERIMENTAL PROCEDURES

### 2.1 Strains and plasmids

Bacterial strains and plasmids are listed in Table 1. *Escherichia coli* strain DH5a (Gibco, BRL) was used as a host for plasmid construction. *E. coli* BL21 (DE3) (Novagen) was used for protein expression. *E. coli* strains were cultured in Luria broth (LB) and grown at 37°C with shaking. *E. faecalis* strains were cultured in M9‐YE or brain‐heart infusion broth (BHI) at 30°C without shaking. Antibiotics were used in the following concentrations: for *E. faecalis*: chloramphenicol, 20 μg/ml; tetracycline, 10 μg/ml; spectinomycin, 1,000 μg/ml; fusidic acid, 25 μg/ml; for *E. coli*: chloramphenicol, 20 μg/ml; kanamycin, 30 μg/ml; carbenicillin, 50 μg/ml. Synthetic **C** and **I** were purchased from New England Peptides.

**Table 1:** Bacterial Strains and plasmids used in this study

Strain or plasmid	Relevant features	Source/reference
*E. coli*		
DH5a	Cloning host	Lab stock
BL21DE3	Expression host	Lab stock
*E. faecalis*		
OG1Sp	Sp^r^	Lab stock
OG1RF	Rif^r^, Fus^r^	Lab stock
DM105	Fus^r^, rpoC::His allele	Lab stock
Plasmids		
pCF10	Tc^r^, conjugative plasmid	(Hirt et al., 2005)
pGEX6p‐1	Carb^r^, protein expression vector	GE Healthcare
pBK2	Cm^r^, shuttle vector	(Kozlowicz, 2005)
pET28‐PrgX	His‐tagged PrgX	(Leonard et al., 1996)
pBK2 + 5	pBK2 carries 5 bp insertion between XBS1 and XBS2 sites	This work
pBK2 + 10	pBK2 carries 10 bp insertion between XBS1 and XBS2 sites	This work

Plasmid pGEX‐PrgX was used to express GST‐PrgX fusion protein and was constructed as following. The *prgX* coding sequence was amplified from pCF10 with primers (prgX‐NdeI GGAATTCCATATGTTTAAGATAGGTTCTGTCC, and prgX‐XhoI CCGCTCGAGGGTACCTCATGACTGCTCTTT TATTT), digested with NdeI and XhoI, and was ligated to pGEX6p‐1 digested with same enzymes.

To construct plasmids pBK2 + 5 and pBK2 + 10, the intergenic region between *prgQ* and *prgX* was excised from plasmids pBKAdd5 and pBKAdd10 and was cloned into pBK2 using enzymes XhoI and BamHI. Plasmids pBKAdd5 and pBKAdd10 (have the 5‐bp and 10‐bp sequence inserted between XBS1 and XBS2 sites (Kozlowicz, 2005).

### 2.2 Protein purifications

GST‐PrgX: *E. coli* BL21 (DE3) cells carrying pGEX‐prgX were grown at 37°C until culture reached an absorbance at 600 nmol L^−1^ of 0.6. Cells were induced with 0.1 mmol L^−1^ of IPTG (isopropyl‐ ‐D‐thiogalactoside) for additional 3 hr at 30°C. Bacterial cells were pelleted by centrifugation at 6400 × g for 10 min and the pellets were resuspended in 10 ml of lysis buffer (20 mmol L^−1^ Tris‐HCl, pH 8.0, 0.15 mol L^−1^ NaCl, 30 mg/ml lysozyme) and sonicated to clarity. Cell lysate was centrifuged at 35,000 × g at 4°C for 20 min. The supernatants were added to a glutathione‐S‐transferase (GST) affinity column. Proteins were incubated with glutathione agarose beads for 40 min, washed extensively with 1 × KPBS buffer. GST tag was cleaved using the PreScission Protease (GE healthcare) following kit protocol. GST tag‐removed PrgX protein was eluted and analyzed by SDS‐PAGE.

HisPrgX was purified following previous protocol, but eluted in different buffers dependent on different applications. For surface plasmon resonance experiments, HisPrgX was eluted in 1 × KPBS buffer with 300 mmol L^−1^ imidazole, then extensively dialyzed with 1 × KPBS buffer. To prevent protein aggregation during dialysis, eluted protein was diluted in 1 × KPBS buffer to a concentration of 0.1–0.2 mg/ml before dialysis.

### 2.3 Electrophoretic mobility shift assays

The LT DNA template containing XBS1 and XBS2 sites (LT DNA, pCF10 sequence from nt 8029 to nt8185 (Hirt et al., 2005)) was PCR amplified from pCF10 using primers as described in. The XBS1 DNA template only has XBS1 site (pCF10 sequence 8029–8131nt) was made by annealing oligos ‐129/‐47‐F (5′TGTTAATATTTTAATTTTAGGTATTGAATACGACACTCGAAGATGTGTTTATTAAGCTATATCCCTTTTTTTTAAAAAAAAATA 3′) and −129/−47R (5′TATTTTTTTTTAAAAAAAAGGGATATAGCTTAATAAACACATCTTCGAGTGTCGTATTCAATACCTAAAATTAAAATATTAACA 3′) to obtain double‐stranded DNA. The XBS2 DNA template only has the XBS2 site (pCF10 sequence from 8109 to 8290; −66 to +114 relative to the *prgQ* transcription initiation site), and was PCR amplified from pCF10 using primers as described in Caserta et al., 2012. In DNA templates with 5‐bp or 10‐bp insertions, sequence GTACC or GTACCTTCTA was inserted between XBS1 and XBS2 sites and at a position of 6‐bp after the XBS1 site. DNA probes were labeled at the 3′‐ends with DIG‐11‐ddUTP using the DIG Gel Shift kit (Roche) by following the manufacturer's instructions. EMSA assays were performed in 20 μl reactions: DIG‐labeled DNA, purified proteins, 1 μg of poly‐[d(A‐T)], 0.1 μg of poly‐l‐lysine, 1 × reaction buffer (20 mmol L^−1^ Tris‐HCl, pH 7.9, 0.1 mol L^−1^ NaCl, 0.1 mmol L^−1^ EDTA, 10% glycerol, 0.01 mol L^−1^ MgCl_2_). The reactions were incubated at room temperature for 15 min and loaded onto 5% polyacrylamide gels in 1 × TBE buffer (Tris, Borate, EDTA, pH 7.9). After electrophoresis at 100 V for 1.5 h, the DNA–protein complexes and DNA probes were electrotransferred onto a nylon membrane (Roche) at 6 V for 2 h using the GENIE electrophoretic blotter (Idea Scientific). DIG‐labeled DNA fragments were visualized by an enzyme immunoassay (DIG Gel Shift Kit, Roche) following the manufacturer's instructions. Note that our previously published EMSA results (Bae, Kozlowicz, & Dunny, 2002; Caserta et al., 2012) demonstrated specific binding of PrgX in the absence of DMF. For the experiments reported here the powdered form of the peptides were initially solubilized in pure DMF at 0.5 mg/ml, and then diluted 10‐fold in KPBS for use as a working stock solution, which was diluted into the EMSA reactions to a final peptide concentration of 40 nmol L^−1^. For the “no peptide controls” equivalent dilutions of DMF with no peptides were added to all reactions involving Apo‐PrgX at a concentration; in the absence of peptides, DMF had no effects on EMSA results.

To estimate protein‐DNA affinity from EMSA assays, the unbound DNA and shifted DNA were quantified using ImageJ software (http://imagej.nih.gov/ij/). Since LT‐DNA has two binding sites, the apparent *K*_d_ values for the binding events were also calculated by applying the two‐site model described in Senear and Brenowitz (1991) using the equations:

$$\theta_{0}=\frac{1}{Z}$$

$$\theta_{1}=K_{1}\frac{\left[L\right]}{Z}$$

$$\theta_{2}=K_{2}\frac{{\left[L\right]}^{2}}{Z}$$

where θ_*i*_ is the fraction of DNA molecules with i proteins bound, [L] is the concentration of protein ligand, *Z* is the binding polynomial equal to 1 + *K*_1_[*L*]+*K*_2_[*L*]^2^ and *K*_1_ and *K*_2_ are the equilibrium association constants. Obtaining the values of θ_*0*_, θ_*1*_, and θ_*2*_ from EMSA, the binding polynomial *Z* was fitted using Curve fitting toolbox in Matlab 2014b to a 2nd degree polynomial (*ax* ^2^+*bx*+*c*) using bisquare robust regression. The constant term of the polynomial (*c*) was forced to value 1 and the other constants were subject to constraints *a*,* b* ≥ 0. Using this method, two binding constants for LT‐DNA‐PrgX binding were obtained. However, since the second band for PrgX‐**C**‐ LT‐DNA and PrgX‐**I**‐LT‐DNA were negligible, θ_*1*_ was assumed to be zero. Hence, the binding polynomial *Z* = 1 + *K*_2_[*L*]^2^ was fitted to obtain the binding constant using the same abovementioned constraints. The plots used for curve fitting to generate the *K*_D_ values presented in results are shown in the Supporting Information.

### 2.4 Gel‐filtration

Size exclusion chromatography experiments were performed using a Superdex 200 Hiload 16/600 column (GE) with a General Electric AKTA FPLC system. Before loading samples, the size column was equilibrated with 2 column‐volume of buffer (20 mmol L^−1^ Tris–HCl (pH 8.0), 300 mmol L^−1^ NaCl). To obtain HisPrgX‐**C** and HisPrgX‐**I** used for size exclusion experiments, nickel affinity columns were first bound with HisPrgX (from 700 ml culture), **C** or **I** (200 μg) dissolved in DMF was then added to the column and incubated at RT for 20 min. After extensive washing, protein‐peptide complex was eluted using buffer: 20 mmol L^−1^ Tris‐HCl, pH 8.0, 300 mmol L^−1^ NaCl, 1 mol L^−1^ imidazole. Affinity purified HisPrgX, HisPrgX‐**C** or HisPrgX‐**I** were loaded to the size exclusion column. The elution was analyzed by monitoring UV absorbance at 280 nmol L^−1^. The column was calibrated using gel‐filtration protein standards (Bio‐rad). Log_10_ (Molecular weight) was plotted against *V*e/*V*o (*V*e is the elution volume at maximum *A*_280_ absorbance for a given sample and *V*o is the void volume of the column determined to be 9.58 ml based on the elution of Dextran blue). The molecular weights of eluted complexes were determined by the elution volumes (*V*e) and the equation from standard curve.

### 2.5 β‐galactosidase assays

*E. faecalis* cultures were grown overnight in M9‐YEG broth at 37°C with selective antibiotics. Cultures were then diluted 1:10 in fresh medium and grown for 90 min at 37°C. For induction, a final concentration of 10 ng/ml of **C** was added and cells were incubated for additional 30 min at 37°C. A modified Miller assay was performed as previously described (Kozlowicz, Bae, & Dunny, 2004).

### 2.6 Determination of binding affinities of C and I for PrgX by surface plasmon resonance

The binding kinetic between the proteins (PrgX) and the peptides cCF10 and iCF10 was measured with surface plasmon resonance using a Biacore T100, using HBS‐P+ supplemented with 30 μmol L^−1^ EDTA and 1% DMSO. The his‐tagged version of PrgX was immobilized onto a NTA Series S sensorchip per manufacturer's specifications. Briefly, for each cycle, the NTA surface was first exposed 1 mmol L^−1^ of NiCl_2_, follow by the his‐tagged protein for immobilization The peptide being analyzed was injected for 600 s (association phase), followed by 900 s of dissociation phase. At the end of the dissociation phase, the surface was then regenerated using 300 mmol L^−1^ EDTA per the manufacturer's instruction. Each peptide was tested at a concentration range of 633–0.6 nmol L^−1^, using twofold serial dilutions, as well as a control run containing no peptide. Examples of SPR binding curves for PrgX/**C** and PrgX/**I** are presented in the Supporting Information.

## 3 RESULTS

### 3.1 Effects of C and I on PrgX binding to the *P*_Q_ promoter region

**Figure 2:**
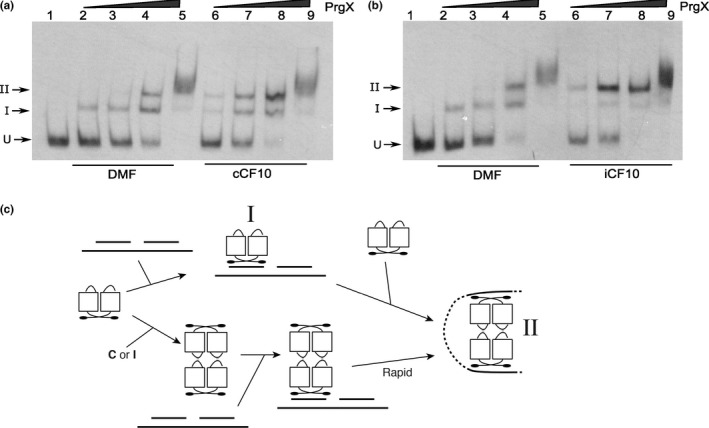
The effects of C and I on PrgX binding to XBS operator sites as determined by mobility gel shift assays. EMSA assays were performed using 8 fmol of digoxigenin‐labeled LT DNA probes with increasing amounts of PrgX protein. PrgX was preincubated for 5‐min at room temperature with 40 nmol L^−1^ of C (a) or I (b) before adding LT DNA. PrgX concentrations: lanes 2 and 6: 38 nmol L^−1^; lanes 3 and 7: 76 nmol L^−1^; lanes 4 and 8: 190 nmol L^−1^; lanes 5 and 9: 568 nmol L^−1^. (c) Cartoon showing the predicted products formed by binding PrgX to LT DNA. The upper part shows stepwise binding of PrgX dimers (Apo‐PrgX) to the XBS1 and XBS2 sites, followed by formation of a DNA loop via interaction between the two dimers, whereas the lower portion shows binding of a preformed tetramer (PrgX‐C or PrgX‐I) to XBS1, followed by very rapid forming of the looped structure. “I” and “II” indicate the shifted and supershifted protein/DNA complexes shown in (a) and (b)

The XBS1 and XBS2 operator‐binding sites were originally identified by DNase I footprinting (Bae et al., 2002), and their importance in regulation of *prgQ* transcription was confirmed using in vitro run‐off transcription assays (Caserta et al., 2012). The XBS1 site is an 11 base pair palindromic sequence, while the XBS2 site only contains half of the palindromic sequence. Apo‐PrgX binds to XBS1 with relatively higher affinity than to XBS2 (Bae et al., 2002). We determined effects of **C** and **I** on PrgX‐DNA binding using in vitro electrophoretic mobility shift assays (EMSAs). As expected, Apo‐PrgX bound to the LT, producing two‐shifted complexes: band I and band II. With increasing concentrations of PrgX, the relative amount of band II increased (Figure 2a, b, lanes 2–5). When either **C** or **I** was added to the reactions at a molar ratio of 1:1 to PrgX, we saw an increased amount of band II relative to the shifts observed at equivalent concentrations of apo‐PrgX (Figure 2a, b, lanes 6–9). The observation that both **C** and **I** increased the supershift was initially surprising, but is consistent with other data described later in this paper. However, at equivalent PrgX concentrations, an increased amount of band II formed in reactions that contained **I** relative to those containing **C** (Compare lanes 6–8 of Figure 2a,b). In control experiments (not shown), we used two other peptides cCAD1 and cPD1, which do not bind to PrgX. These peptides had no effect on the PrgX‐DNA‐binding profiles^.^ As illustrated in Figure 2c, Band II likely results from assembly of a DNA‐bound tetramer, either by sequential binding of two dimers to the XBS sites and loop formation via protein/protein interactions, or by binding of a preformed tetramer to XBS1 and subsequent loop formation and binding of the tetramer to XBS2. Based on additional results described below, we suspect that band II formation by peptide/PrgX complexes occurs primarily by the latter pathway, especially in the presence of **I,** while the former pathway is active in the absence of peptides.

### 3.2 An essential role for DNA looping in PrgX regulation of *P*_Q_ is confirmed by analysis of effects of altering the spacing between XBS1 and XBS2

**Figure 3:**
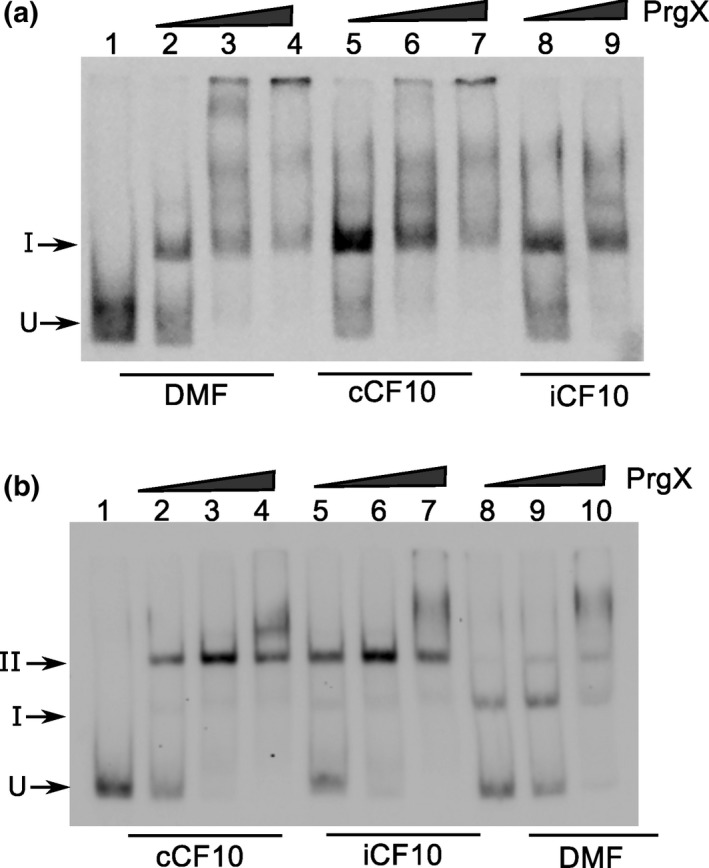
PrgX binds differently to sequences that have 5 or 10 bp inserted between two operators. EMSA experiments were performed as described in Figure 2. Purified PrgX protein concentrations used in the experiment: lanes 2, 5, 8: 10 nmol L^−1^; lanes 3, 6, 9: 25 nmol L^−1^; lanes 4, 7, 10: 100 nmol L^−1^. PrgX was preincubated with DMF, C or I for 5 min before addition of probes. (a). DNA probe has 5 bp inserted between XBS1 and XBS2 sites. (b). DNA probe has 10 bp inserted between the two binding sites. EMSA experiments were performed as described above

Previous results (confirmed in this paper) identified two operator sites for PrgX binding in the region upstream from the *prgQ* transcription start site (Bae et al., 2002). The spacing between the two XBS operator sites, and the cooperative binding of PrgX to these operators (Bae et al., 2002), suggested that PrgX repression of *P*_Q_ involves DNA looping. The distance between centers of XBS1 and XBS2 sites is 91‐bp, placing the two sites on the same face of the DNA double helix. We confirmed the role of DNA looping using a helical‐twist experiment. Adding 5‐bp (half a turn of the double helix) between two operator sites places the two sites on opposite faces of the double helix, likely decreasing loop formation and reducing XBS2 binding. Insertion of a 10‐bp spacer should restore the two binding sites to the same face of the DNA helix, and restore loop formation. We tested the effects of inserting 5‐ or 10‐bp spacers between XBS1 and XBS2 on DNA binding in vitro. Apo‐PrgX binding to the +5 probe shifted the probe nearly completely to band I, with no distinct supershifted band II (Figure 3a, lanes 2–4). With the +10 probe, the Apo‐PrgX binding profile was similar to LT DNA wild‐type probe (compare Figure 3b, lanes 8‐10 to Figure 2a,b lanes 2–4), and addition of either **C** or **I** peptides to EMSAs containing the +10 probe increased the supershift to band II, similar to the results obtained with the wild‐type probe (Figure 2). However, with the +5 probe, the presence of the peptides produced only a minimal shift to band II (Figure 3a, lanes 5–9). These EMSAs and our previous studies (Kozlowicz et al., 2006; Shi et al., 2005) support a model where the repressing structure resulting from PrgX binding to pCF10 DNA is comprised of two interacting PrgX dimers, with each bound to one XBS site, and the intervening DNA forming a loop (as illustrated in Figure 1i, and see subsequent results).

**Figure 4:**
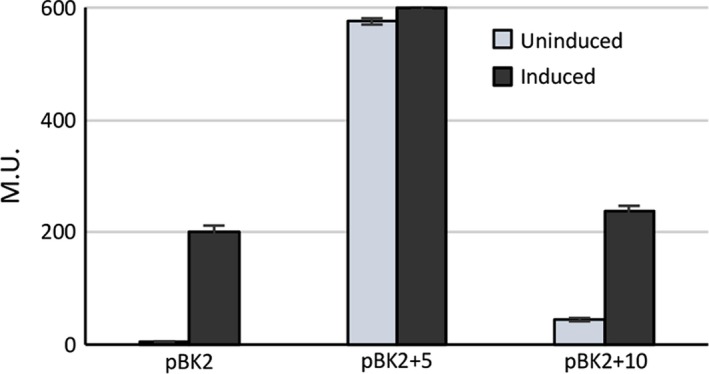
Expression of β‐galactosidase from *E. faecalis* cells containing pBK2, pBK2 + 5, and pBK2 + 10. Plasmids pBK2 + 5 and pBK2 + 10 contain 5 or 10 bp inserted between the XBS1 and XBS2 sites. Reporter constructs were transformed in OG1RF/pCF10. Overnight *E. faecalis* cells containing reporter constructs were diluted 1:10, grown to log phase and then either left uninduced or induced with 10 ng/ml of C. Data from one experiment representative of three repeats

To examine the effects of looping in vivo, we introduced the +5 and +10 base pair insertions between XBS1 and XBS2 sites on the reporter plasmid pBK2, which has a pheromone‐inducible *lacZ* reporter gene fused downstream from *P*_Q_. This enables transcription from *P*_Q_ to be monitored by measuring β‐galactosidase activity in cell extracts. We transformed these constructs into either OG1Sp or OG1Sp/pCF10 (to provide PrgX protein in trans) and the resulting strains were assayed for β‐galactosidase production in the presence or absence of **C**. The five base pair insertion construct displayed a totally de‐repressed phenotype that was insensitive to **C** (Figure 4). Providing PrgX in trans from pCF10 had no effect on *lacZ* activity with this construct. In the absence of PrgX, the 10‐bp spacer mutation showed a de‐repressed phenotype (Figure 4). However, when PrgX was provided in trans by pCF10, β‐galactosidase expression was repressed in this construct (Figure 4). Addition of **C** to pCF10/pBK2 + 10 cultures induced *lacZ* expression, confirming that the +10 spacer mutation allowed for **C**‐sensitive *P*_Q_ regulation, similar to wild type.

### 3.3 PrgX and RNAP compete for binding to the *P*_Q_ promoter

**Figure 5:**
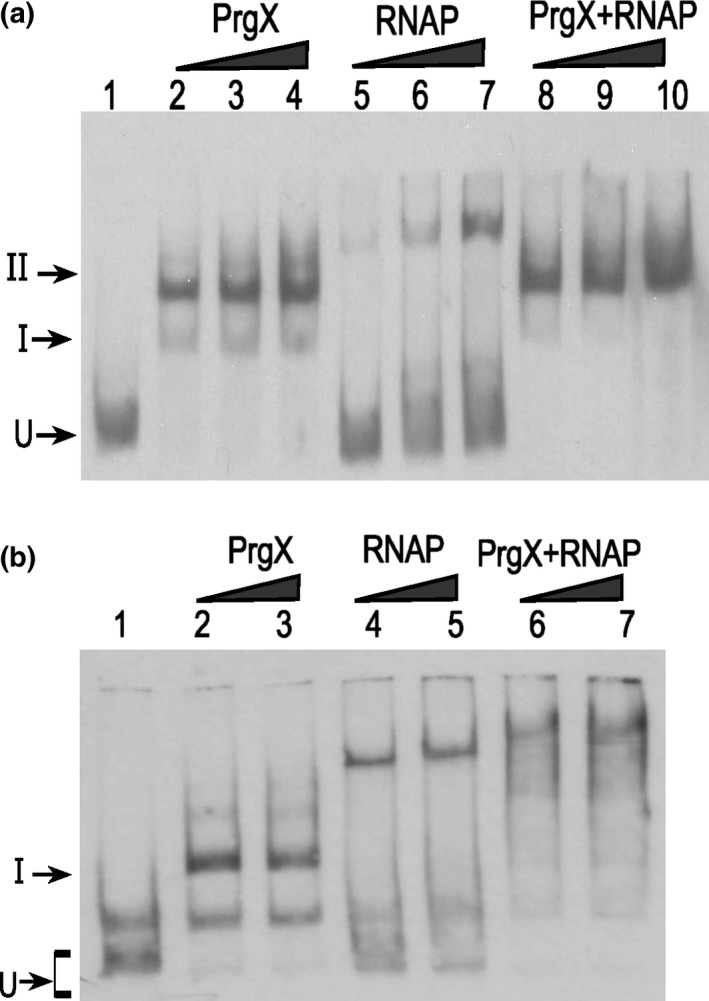
PrgX and *E. faecalis* RNA polymerase (RNAP) compete for binding at *prgQ* promoter. Electrophoretic mobility shift assays were performed using 8 fmol of digoxigenin‐labeled DNA probes and various amounts of purified PrgX and RNAP. PrgX binds to DNA probe containing both operator XBS1 and XBS2 (LT template) and forms two‐shifted complexes (band I and II). “U” indicates the position of unbound probe. The components in each lane are as indicated in the figure. (a). PrgX hinders RNAP binding to *prgQ* promoter. LT DNA was first incubated with RNAP for 10 min at RT before addition of PrgX to the reactions. Untagged PrgX was cleaved from GST‐PrgX. PrgX concentration used: lanes 2 and 8: 19 nmol L^−1^; lanes 3 and 9: 38 nmol L^−1^; lanes 4 and 10: 72 nmol L^−1^. RNAP concentration used: lanes 5 and 8: 60 nmol L^−1^; lanes 6 and 9: 70 nmol L^−1^; lanes 7 and 10: 150 nmol L^−1^. (b). PrgX and RNAP form a stable complex on LT DNA which has mutations in the XBS2 site. In these reactions, PrgX and RNAP were incubated with probe DNA probes at RT for 15 min. PrgX concentration used: lanes 2 and 6: 18 nmol L^−1^; lanes 3 and 7: 54 nmol L^−1^. RNAP concentration used: lanes 4 and 6: 200 nmol L^−1^; lanes 5 and 7: 240 nmol L^−1^

Since XBS2 is between the −35 and −10 regions of *P*_Q_, a simple model for PrgX repression would be via inhibition of RNA polymerase binding to *P*_Q_ by steric hindrance in the XBS2 region. We used EMSAs to determine the effects of PrgX on RNA polymerase (RNAP) binding to P_Q_. EMSAs were performed using purified PrgX, RNAP, and the LT DNA probe (a segment of pCF10 DNA containing both XBSs). PrgX and RNAP each bound to LT DNA, showing different shifted complexes on native polyacrylamide gels. In Figure 5a, lanes 2–4 show that PrgX interactions with LT result in the shifted bands I and II, reproducing the results shown in Figure 2. RNAP bound to LT DNA and formed a more slowly migrating band (Figure 5a, lanes 5–7). When both PrgX and RNAP were added to LT DNA, formation of the high‐molecular‐weight RNAP‐DNA species was nearly eliminated and PrgX/DNA complexes corresponding to Bands I and II were observed that were very similar to those obtained in the absence of RNAP (Figure 5a, lanes 8–10). No very slowly migrating species indicative of large complexes containing both PrgX and RNAP were observed, even at very high protein concentrations. This indicates that simultaneous binding of PrgX and RNAP to the *P*_Q_ region did not occur under these conditions.

We then used a variant DNA probe where the XBS2 sequence was changed (B2 m1) in EMSAs; this change did not affect XBS1, or the −35 or −10 regions of the *prgQ* promoter, but was shown to reduce PrgX binding to XBS2 and also reduced PrgX repression in run‐off in vitro transcription assays (Caserta et al., 2012). Binding of PrgX to the B2 m1 probe produced the band I shift, however, there was a dramatic reduction in band II (Figure 5b, lanes 2–3). The band shift was observed when RNA polymerase to this probe was very similar to that of the wild‐type LT, indicating that the B2 m1 mutation did not affect polymerase binding to *P*_Q_ (Figure 5b, lanes 4–5). When both PrgX and RNAP were added to B2 m1 DNA, there were supershifted bands formed in EMSAs, indicative of complexes containing both PrgX and RNAP (Figure 5b, lanes 6–7). These cumulative results indicate that PrgX precludes access of RNAP to *P*_Q_ in the context of a wild‐type XBS2, but RNAP can bind to *P*_Q_ on DNA probes concurrently bound to PrgX (via XBS1) if the XBS2 sequence is mutated to reduce PrgX binding at that site.

### 3.4 Affinities of C and I for PrgX and their effects on PrgX oligomerization

Using surface plasmon resonance, the binding kinetics and affinities of **C** and **I** to PrgX were obtained. Both peptides bound to PrgX with similar kinetics and high affinities: the dissociation constant (*K*_D_) for PrgX‐**C** is 6.856 × 10^−13^mol L^−1^, and for PrgX‐**I** is 1.52 × 10^−13^mol L^−1^ (Table 2); examples of binding/dissociation curves used to calculate these values are presented in the Supporting Information. The extremely low dissociation constants for both peptides, combined with the low intracellular concentrations of the free peptides present under normal physiological conditions, make it very unlikely that changes in the induction state of donor cells result from replacement of one peptide with the other in DNA‐bound PrgX/peptide complexes.

**Table 2:** PrgX binding kinetics and affinities to peptides C and I

Protein‐peptide	*K*_a_ (mol L^−1^s^−1^)	*K*_d_ (s^−1^)	*K*_D_ (mol L^−1^)
PrgX‐**C**	8.02E+7	5.50E‐5	6.86E‐13
PrgX‐**I**	1.34E+8	2.09E‐5	1.52E‐13

Values were obtained using Biacore T100 system. *K*_a_ and *K*_d_ were determined from binding and dissociation curves and K_D_ was calculated by dividing *K*_d_ by *K*_a_.

**Figure 6:**
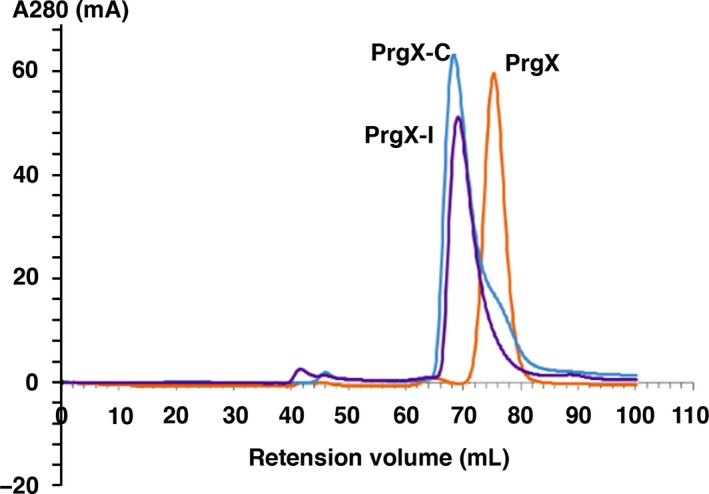
Peptide‐induced PrgX oligomerization increase in solution. PrgX, PrgX‐C, and PrgX‐I complexes were purified using Ni‐affinity chromatography. Purified protein oligomer status were analyzed using size exclusion chromatography. The UV absorbance at 280 nmol L^−1^ is plotted against elution volumes. 1(orange):PrgX; 2(purple): PrgX‐I; 3(blue): PrgX‐C

Genetic and biochemical experiments indicated that PrgX forms dimers in vivo (Bae & Dunny, 2001; Kozlowicz et al., 2004), and structural analysis suggested that DNA‐bound tetramers could be the functional repressing forms of PrgX (Kozlowicz et al., 2006; Shi et al., 2005). However, the oligomerization state of PrgX/peptide complexes in solution is unknown. We used size exclusion chromatography to determine PrgX oligomerization states in solution (in the absence of DNA). Apo‐PrgX eluted as a dimer with an apparent mass of 74 KDa (Figure 6). We purified **C**‐ or **I**‐bound PrgX by Ni^++^ affinity chromatography and then subjected the complexes to size exclusion chromatography. PrgX‐**C** and PrgX‐**I** both eluted at apparent masses of 140‐145 KDa (Figure 6), consistent with tetramers. To exclude nonspecific effects caused by peptides, the cAD1 peptide (the inducer of conjugation in the pAD1 system), which does not interact with PrgX, was used as a control. PrgX exposed to cAD1 eluted at the same position as apo‐PrgX (not shown), confirming that stable PrgX tetramers are specifically generated by both **C** and **I**.

### 3.5 High Binding affinities of both PrgX‐C and PrgX‐I tetramers for the *P*_Q_ promoter region

**Figure 7:**
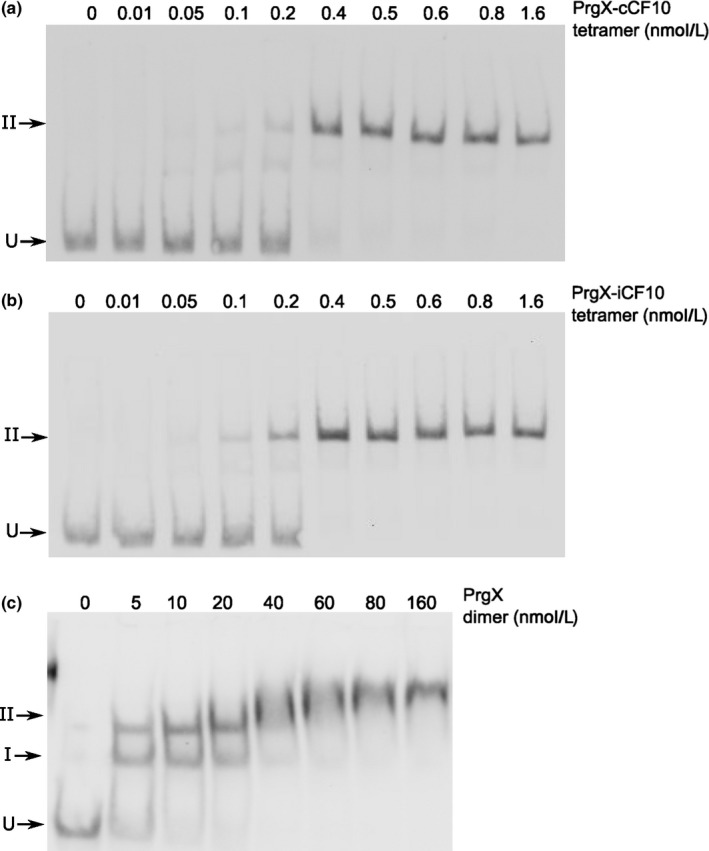
Binding of purified PrgX (c) dimers, PrgX‐C (a), or PrgX‐I (b) tetramers binding to LT DNA. EMSA assays were performed using 8 fmol of digoxigenin‐labeled LT DNA probes and increasing concentrations of PrgX proteins. Protein concentrations used are as indicated in the figure

We collected PrgX‐**C** and PrgX‐**I** tetramer fractions from size exclusion columns and added these purified tetramers to the EMSAs using the LT probe containing both XBSs. For both tetramers, there was a nearly complete supershift to band II at extremely low PrgX concentrations (Figure 7a, b); the supershifted species in this experiment were equivalent to band II in Figures 2, 3, but the use of purified peptide‐tetramers in the experiment shown in Figure 7 eliminated requirement for peptide binding to PrgX and tetramer formation prior to DNA binding.

Since the use of purified peptide/PrgX tetramers essentially converted the formation of the band II complex to a single‐step, we measured the densitometry of free and protein‐bound DNA at different protein concentrations to determine the binding affinities of PrgX‐**C** and PrgX‐**I** for LT, as described in the Methods. Based on binding polynomial fitting (curves shown in Supporting Information), the calculated K_D_ of both Peptide/PrgX tetramers were <1 nmol L^−1^, the calculated *K*_D_ of PrgX dimer to XBS1 site was about 10‐fold higher and the *K*_D_ of PrgX dimers bound to XBS2 nearly 1000‐fold higher (Table 3).

**Table 3:** Equilibrium dissociation constants of PrgX, PrgX‐I, and PrgX‐C binding to LT‐DNA

Protein‐DNA	*K*_D_ (nmol L^−1^)
PrgX‐**C**‐LT DNA	*K* = 0.43 ± 0.10
PrgX‐**I**‐LT DNA	*K* = 0.21 ± 0.052
PrgX‐LT DNA	*K*_xbs1_ = 4.04 ± 3.36
	*K*_xbs2_ = 341.85 ± 377.5

Equilibrium dissociation constants were estimated from binding polynomials presented in the Supporting Information. Values are the mean ± SD of three independent EMSA experiments similar to those depicted in Figure 7. PrgX binding to LT produced two shifts, with *K*_xbs1_ being the *K*_D_ of binding to XBS1 site, and *K*_xbs2_ is the *K*_D_ of binding to XBS2 site. Binding of PrgX‐**C** or PrgX‐**I** tetramers to LT produced a single shifted species, which corresponds to the second shift observed with Apo‐PrgX.

### 3.6 Effects of peptides on binding to DNA probes containing single XBSs

**Figure 8:**
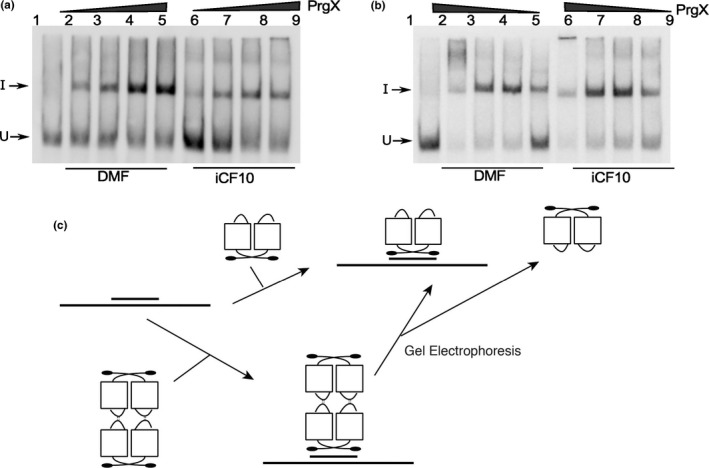
Binding of Apo‐PrgX and PrgX/I to DNA only containing one operator sequence. EMSA assays were performed using 8 fmol of digoxigenin‐labeled LT DNA probes with increasing amounts of protein. PrgX was preincubated with DMF or I for 5 min before addition of probes. (a) XBS1 DNA template has only the XBS1 binding site. PrgX concentration used: lanes 2–5 and lanes 6–9: 10, 25, 100, and 200 nmol L^−1^, respectively. (b) XBS2 DNA template has only the XBS2 binding site. PrgX concentration used: lanes 2–5 and lanes 6–9: 200, 100, 25, and 10 nmol L^−1^, respectively. (c) Binding PrgX to DNA contains a single‐binding site illustrated by cartoon. Gel electrophoresis dissociated PrgX‐C or PrgX‐I tetramers, resulting in band shifts similar to those produced by PrgX dimers

Previous studies suggested reduced binding of apo‐PrgX to DNA probes lacking XBS1 and virtually no supershifted species resembling Band II (Bae et al., 2002), but these studies did not examine effects of **C** or **I**. We thus examined the effects of the peptides on PrgX binding to DNA templates that only contain one binding site; Figure 8 shows effects of **I**, while a parallel experiment examining effects of **C** gave virtually identical results (Supporting Information). Two probes were tested, one containing only the XBS1 site (Figure 8a), and the other containing only XBS2 (Figure 8b), and we added either **I**, or no peptide to PrgX/DNA‐binding reactions. Single shifted bands of the same mobility were observed for all reactions. Based on our cumulative results, we conclude that all the shifted species in Figure 8 represent the DNA probes bound to a dimer. It is likely that the gel electrophoresis conditions promote dissociation of tetramers into dimers in the absence of a second XBS site on the DNA probe. In the case of the EMSAs shown previously (Figures 2, 7), the increased stability of the looped complexes resulting from multiple protein/DNA interactions likely prevented tetramer dissociation in the gels.

## 4 DISCUSSION

This study sought to identify how specific interactions of the **C** and **I** signaling peptides with the master regulator PrgX modulate expression of pCF10 conjugation genes in *E. faecalis*. The regulatory circuitry of the pCF10 system is complex and multiple transcriptional and posttranscriptional mechanisms are required for normal control (Dunny, 2013). While many of these mechanisms have been characterized, we lack full understanding of the dynamic process by which they function coordinately to convert a pCF10‐containing cell from the uninduced to the induced state, and to return the cell to the uninduced state following a mating response. The induction status of cells carrying pCF10 is determined by the molar ratio of **I** to **C** in the donor cytoplasm, following their import from the growth medium. Genetic, biochemical, and structural studies all suggested that the **C** and **I** peptides function via direct binding to PrgX; reviewed in (Dunny, 2013). While both peptides bind to a same pocket in PrgX, they induce different conformations in PrgX‐C‐terminus (Kozlowicz et al., 2006; Shi et al., 2005). However, no structural changes in the PrgX N‐terminal DNA‐binding domain result from peptide binding, suggesting an indirect mechanism by which PrgX function is modulated by the peptides.

Structural and genetic analyses (Kozlowicz et al., 2006; Shi et al., 2005) suggested that the repressing structure of PrgX bound to the upstream regulatory region of *prgQ* was a PrgX**/I** tetramer each dimer bound to one XBS operator site. These two operator sites were connected by a DNA loop stabilized by protein/protein interactions between the bound PrgX dimers (Figure 1i). Interactions between pairs of PrgX dimers were predicted to be enhanced by a 10 amino acid loop near the carboxy terminus whose structure is stabilized by binding of **I** to PrgX (Kozlowicz et al., 2006). At the DNA level, overlap between XBS2 and *P*_Q_ would result in repression by steric hindrance of RNA polymerase binding to *P*_Q_ when XBS2 was occupied by PrgX.

The present data confirm several important features of previous working models. We obtained both in vitro (Figure 3) and in vivo (Figure 4) data for the previously predicted requirement for DNA looping in PrgX regulation of conjugation (Kozlowicz, 2004, Kozlowicz et al., 2006). The results of the helical‐turn experiments presented here (Figures 3, 4) provide strong evidence of an essential role for looping, and further highlight the importance of interactions between PrgX dimers bound to each XBS. The results also confirm that PrgX repression results from exclusion of RNA polymerase binding to *P*_Q_ via PrgX‐mediated steric hindrance in the XBS2 region (Figure 5).

DNA looping is an important transcriptional regulatory mechanism in both prokaryotes and eukaryotes. However, only a few such systems have been characterized in detail (Cournac & Plumbridge, 2013). In *E. coli*, there are six operons well studied and experimentally shown to be regulated by a DNA loop (*ara*,* lac*,* gal*,* nag*,* deo*, and *pstG*). Recently, Ramachandran et al. (Ramachandran et al., 2014) reported the existence of a DNA loop in the conjugative pLS20 plasmid of the soil bacterium *Bacillus subtilis*. Similar to pCF10, transcriptional regulation of pLS20 major conjugation operon is repressed by the master repressor protein Rco_LS20_. Rco_LS20_ controls two overlapping divergent promoters by binding to two operator sites simultaneously. Rco_LS20_ mediated DNA loop formation was demonstrated by helical‐turn spacing experiments. However, unlike PrgX, Rco_LS20_ was found to form tetramers in solution in the absence of modulating peptide cofactors. The DNA loops found in pCF10 and pLS20 are the shortest loops identified to date in nature; the presence of poly‐A and –T tracts in the intervening regions probably serves to enhance looping by DNA bending.

Binding affinities of signaling peptides for their cognate receptors have not been previously measured for any of the enterococcal pheromone systems. Since multiple in vivo experiments have shown that excess **I** is required for inhibition of **C** (reviewed in (Clewell, Francia, Flannagan, & An, 2002; Clewell et al., 2014), we expected that the binding affinity of **C** for PrgX would be stronger than that of **I**, and that induction could result from replacement of **I** with **C** in preformed PrgX oligomers (Figure 1i , ii), or from conversion of apo‐PrgX oligomers to PrgX/**C** oligomers. The lack of the stable carboxy‐terminal loop in PrgX/**C** complexes was predicted to favor dissociation of tetramers to dimers in the donor cell cytoplasm, leading to dissociation of PrgX from XBS2, and allowing RNA polymerase to access *P*_Q_ (Figure 1ii); similarly, shut down of the pheromone response could occur via a reversal of the induction process, where the high ‐levels of **I** produced during induction would eventually displace bound **C** from PrgX complexes. The new results reported here necessitate refinements of the working model to accommodate a more accurate picture of the binding interactions of the **C** and **I** peptides with PrgX, and of the effects of these peptides on PrgX oligomerization state and function.

In this study, we made the striking observation that the binding affinities of both peptides for PrgX are extremely high, with *K*_D_ values approaching 10−^13 ^mol L^−1^ (Table 2). Given that **I** must be added in excess to inhibit the response of donor cells to **C**, we expected that **C** would have higher affinity for PrgX. In fact **I** shows slightly stronger binding, and affinities for both peptides are extremely strong indicating that peptide/PrgX binding is essentially irreversible in vivo. Thus, in donor cells, one peptide is highly unlikely to replace the other in a preformed PrgX complex. Furthermore, addition of either peptide to PrgX in solution converted the dimer form to tetramers, which could be stably purified by size exclusion chromatography (Figure 6). These findings also beg the question of the mechanism by which **C** and **I** compete. This suggests that the functional competition between the two peptides occurs during PrgZ/Opp‐mediated import of the peptides. Recently, the structure of the secreted lipoprotein PrgZ and its complexes with **I** and **C** were analyzed (Berntsson, Schuurman‐Wolters, Dunny, Slotboom, & Poolman, 2012). The results indicate that PrgZ/**C** complexes are more stable than PrgZ**/I** complexes. This is consistent with the idea that excess **I** is required in the medium in order to achieve a sufficient intracellular peptide concentrations to change the induction state of donor cells.

EMSA analysis (Figure 2, 3, 7) supported the notion that both PrgX/peptide complexes produced a supershifted protein/DNA complex consistent with a tetramer bound to the probe DNA. PrgX/**I** complexes induced complete supershifting at slightly lower protein concentrations than PrgX/**C.** Either peptide complexed to PrgX generated the supershift at much lower concentrations than apo‐PrgX (compare EMSA results in Figures 2, 7). The results presented in this paper suggest that the differences in the repressed versus induced forms of PrgX/XBS DNA may relate to (subtle) structural differences between the **C**‐X tetramer/DNA complexes and **I‐**X tetramer/DNA complexes (Figure 1, part i. vs. iii.). Our previous analysis of pCF10 in combination with the new results reported here suggest a model for regulation where the relative concentrations of three different operator DNA/PrgX complexes ultimately determine the induction state of donor cells. In complexes i and iii of Figure 1, a PrgX tetramer is bound to the two operator sites, and both DNA‐protein and protein–protein interactions contribute to complex stability. In the case of PrgX/**I** complexes with DNA, all of the proteins should be aligned within the plane of the illustration (Figure 1i), based on structures of the protein/peptide complexes (Kozlowicz et al., 2006; Shi et al., 2005). In contrast, the tetramers of PrgX/**C** complexes are distorted (Shi et al., 2005), such that the one pair of dimers is rotated out of the plane of the figure shown in Figure 1iii. This distortion places torsional stress on the DNA loop, likely decreasing overall stability of the complex structure. Because the weak link in the complex is the binding interaction between PrgX and XBS2, we suggest that this distorted structure would be less able to compete with RNA polymerase for binding in the XBS2 region. While it may seem surprising that this subtle structural difference could explain peptide‐mediated induction, we note that the direct effect of addition of **C** to a donor culture on transcription initiation from *P*_Q_ is actually very modest, in the range of two‐ to fourfold (Caserta et al., 2012). This very small difference is greatly amplified (~100‐fold) by multiple posttranscriptional mechanisms that control elongation of transcription from *P*_Q_ into the conjugation genes, as well as the synthesis of *prgX* mRNA and additional sRNA regulators produced from the convergent *prgX* promoter located in the 5′ terminal region of the *prgQ* operon (Chatterjee et al., 2011; Johnson et al., 2011). On the other hand, we also observed that in EMSA assays involving PrgX/**C**, slightly more of the singly shifted probe is detected relative to PrgX/**I** complexes at equivalent protein concentrations. This could indicate some dissociation of PrgX/**C** tetramers. Thus, structural differences in the two tetramer‐bound complexes, and increased tetramer dissociation in the case of PrgX/**C** could both contribute to a decrease in the ability of PrgX**/C** to compete with RNA polymerase for binding in the XBS2 region. Footprinting analysis of the various PrgX/peptide/DNA complexes, as well as examination of the ability of purified PrgX/**C** versus PrgX/**I** tetramers to inhibit *prgQ* transcription in vitro could help resolve these models.

It is interesting to consider transcription regulation in the pCF10 system by PrgX in relation to other well‐studied bacterial transcription factors. Many of the canonical transcription factors such as LacI are present in very low concentrations, and are modulated by low molecular weight ligands (co‐repressors, etc.) that are generally more abundant (Muller‐Hill, 1998). In contrast, wild‐type *E. faecalis* cells carrying pCF10 contain a large excess of PrgX (~15‐fold ratio of X dimers/XBSs) (Caserta et al., 2012), while the extracellular (and probably intracellular) concentrations of the peptides are very low (Mori et al., 1988; Nakayama et al., 1994). Although apo‐PrgX can bind DNA (Figure 2), and repress *prgQ* transcription in vitro (Caserta et al., 2012), the results of the EMSA experiments reported here indicate that PrgX bound to either peptide produces shifted and supershifted complexes with its DNA target at much lower protein concentrations than apo‐PrgX. This suggests that upon import of either peptide, any existing apo‐PrgX/DNA complexes would be rapidly replaced by peptide‐containing complexes. The affinities of both peptide complexes for DNA are strong (both in the nmol L^−1^ range, Table 3), but not as strong as the peptide‐binding affinities for PrgX. Thus, we expect that changes in the ratio of one type of peptide‐containing DNA‐bound complex to the other can result from occasional dissociation of the bound protein from the DNA, from synthesis of new XBSs during plasmid replication, or from protein turnover. In other words, changes in donor induction state likely result from changes in the type of PrgX complex bound to operator sites rather than swapping one peptide for another in a preexisting complex. The refinements of the stepwise model for pheromone induction described here may affect some of the parameters and assumptions necessary for more accurate mathematical modeling of the pheromone response (Chatterjee et al., 2011, Chatterjee et al., 2013).

## CONFLICT OF INTEREST

None of the authors has a conflict of interest regarding this research.
