# Supplementary material for: Mechanisms of peptide sex pheromone regulation of conjugation in Enterococcus faecalis
Source: Microbiologyopen. 2017 May 19;6(4):e00492. doi: 10.1002/mbo3.492 (PMC5552905; doi:10.1002/mbo3.492)

Supplementary Information for Chen et al., "Direct Effects of Peptide Sex Pheromones…"

Supplementary Figure 1. Binding and dissociation curves of I and C peptides to PrgX as determined by Biacore Surface plasmon resonance. Solutions of **I** or **C** peptides at the indicated concentrations (nM) were flowed over a bound sample of PrgX, as described in the methods. A. Sample binding curve for **C.** B. Sample binding curve for **I**. The complete raw data file from these experiments is available from the corresponding author on request.

S1A.

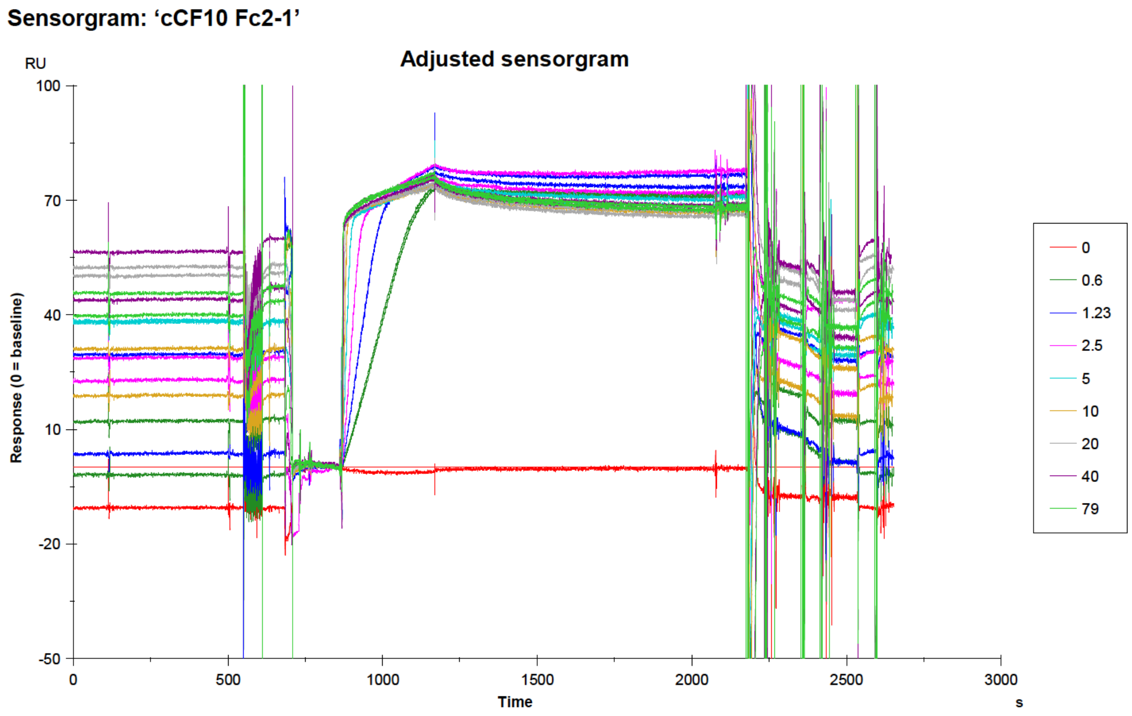

S1B.

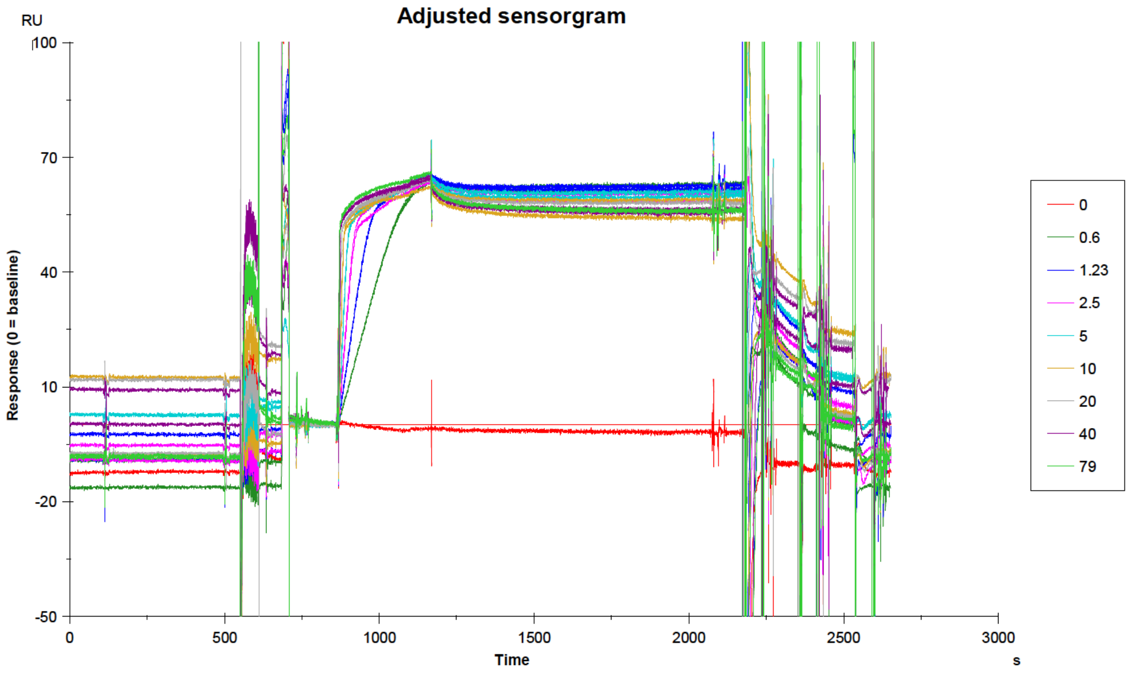

Adjusted sensorgram
RU
100
70
40
10
-20
-50
Response (0 = baseline)
0
500
1000
1500
2000
2500
3000
Time
s
0
0.6
1.23
2.5
5
10
20
40
79

Supplementary Figure 2. Binding polynomial fitting used to calculate K_D values for PrgX binding to XBS sites of pCF10. As described in the main text, densitometric analysis of EMSAs similar to those shown in Fig. 7 were used to generate curves.

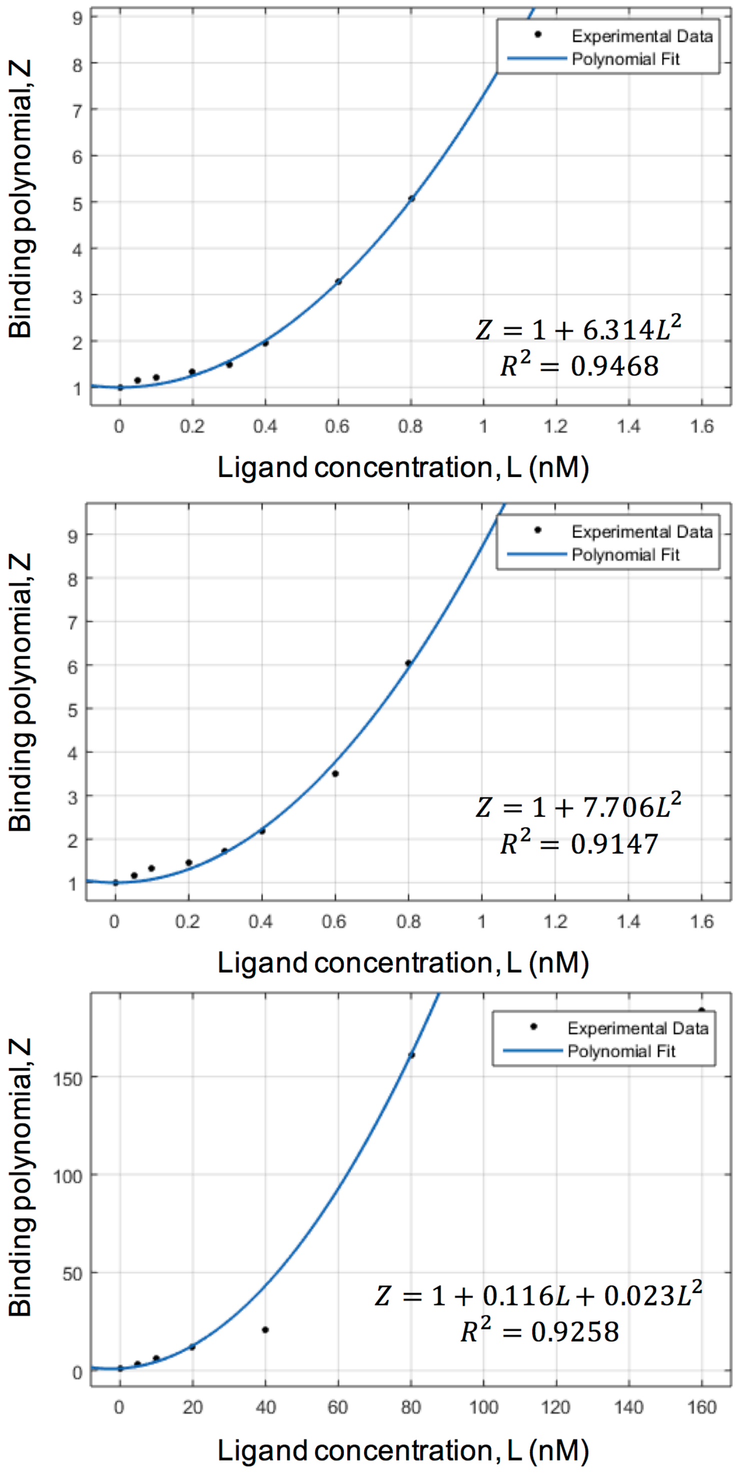

<u>Figure S3.</u> EMSA assays were performed using 8 fmol of digoxigenin-labeled LT DNA probes with increasing amounts of protein. PrgX was pre- incubated with DMF and cCF10 for 5 minutes before addition of probes. **(A)** DNA template has only the XBS1 binding site. Lanes 1 and 5 are probes alone. PrgX concentration used: lanes 2 to 4 and lanes 6 to 8: 10, 25, and 100 nM respectively. **(B)** DNA template has only the XBS2 binding site. Lane 1 is the probe only. PrgX concentration used: lanes 2 to 5 and lanes 6 to 9: 10, 25, 100, and 200 nM respectively. Compare to Figure 8 of main text.

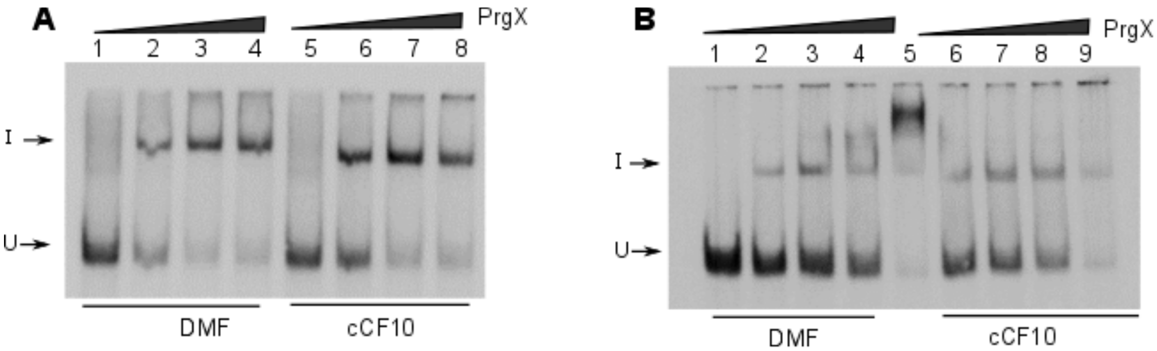

Supplement: Supplementary file 1 [file MBO3-6-na-s001.pdf]
